# Atypical symptoms of malignant hyperthermia: A rare causative mutation in the RYR1 gene

**DOI:** 10.1515/med-2021-0396

**Published:** 2022-02-02

**Authors:** Qiao Ling Wang, Yu Fang, Shuo Guo Jin, Jing Tao Liang, Yi Feng Ren

**Affiliations:** Department of Ministry of Science, Hospital of Chengdu University of Traditional Chinese Medicine, Chengdu, Sichuan, 610072, China

**Keywords:** anesthesia, type 1 ryanodine receptor, mutation, malignant hyperthermia, atypical symptoms, case report

## Abstract

Malignant hyperthermia (MH) is an autosomal dominant genetic condition of the skeletal muscle triggered by inhaled general anesthetic agents or succinylcholine and associated with a hypermetabolic state and skeletal muscle rigidity. Tachycardia, increased carbon dioxide production, hypercarbia, hyperthermia, acidosis, hyperkalemia, cardiac arrhythmias, muscle rigidity, and rhabdomyolysis are common symptoms of MH. As the progression of the syndrome could be rapid or less evident, even experienced physicians have difficulty in diagnosing MH, which can lead to delays in treatment and increased mortality. We report a rare case of a 36-year-old man, who underwent open reduction and internal fixation of the left clavicle after inhaled anesthetics. The patient developed dyspnea, hypotension, unremitting hyperthermia, tachycardia, and elevated serum myoglobin, and finally died of pyemia and disseminated intravascular coagulation. We reviewed the process of disease development, summarized the steps of diagnosis, and improved genetic testing. Exome sequencing revealed a new mutation c.8519G>A (p.arg2840 GLN) in the RYR1 gene that could be associated with MH. The gene mutation was also found in his daughter’s genetic test. This case emphasized the importance of the awareness of MH and its atypical clinical symptoms. The presence of dyspnea, hypotension, unremitting hyperthermia, tachycardia, and raised myoglobin in serum might further strengthen the clinical diagnosis of suspected MH.

## 1 Introduction

Malignant hyperthermia (MH) is an autosomal dominant genetic condition of the skeletal muscle, which is susceptible to inhaled general anesthetic agents or succinylcholine, manifesting as a hypermetabolic state and skeletal muscle rigidity [1,2,3]. Dangerously high body temperature, abnormal heart rhythm, increased carbon dioxide production, increased acidity in the blood, high blood potassium, muscle rigidity, rhabdomyolysis, and renal failure are the typical symptoms of MH [4]. Rarely, in humans, the stressors inducing MH may include vigorous exercise and heat [5]. The exact incidence of MH is unknown, but is estimated at 1:200–1:250,000 [1], and MH reactions occur in 1:10,000–1:250,000 of anesthetic procedures [2,6,7]. The likely risk factors include a family history of MH and a history of masseter rigidity with anesthesia use [1,2]. MH occurrence is associated with a high mortality rate if it cannot be recognized immediately and diagnosed earlier. Timely treatment using dantrolene sodium, a muscle relaxant that inhibits the release of Ca^+^ from the endoplasmic reticulum by acting on RYR1 [8], can reduce the mortality from 80% in the 1970s to <5% nowadays [1,2].

Clinical episodes of MH syndrome have been associated with at least 210 mutations in the type 1 ryanodine receptor (RYR1) gene and four mutations in the dihydropyridine calcium channel receptor gene. Nevertheless, among the more than 400 known RYR1 variants, only 42 RYR1 and 2 CACNA1S variants are confirmed as the causative gene mutations for MH and can be used in diagnostic genetic testing for MH [2,9].

In this study, we report a novel mutation in the RYR1 gene that is probably associated with MH encountered in our clinical practice. The awareness of the occurrence of MH during anesthesia should be emphasized.

## 2 Case presentation

A 90 kg, 36-year-old Chinese (Han ethnicity) man presented with left clavicle pain and was admitted to the Department of Orthopedic ICU, the Affiliated Hospital of Chengdu University of Traditional Chinese Medicine on March 20, 2018. The patient’s injuries were sustained from a traumatic accident one day prior to the admission, but he did not seek medical attention immediately. Then, he was deemed a candidate for open reduction and internal fixation for a fracture of the left clavicle.

The patient was in good health and had no history of common chronic diseases, infectious diseases, major trauma, surgery, blood transfusion, and food or drug allergy. He had no family history of medical conditions, and his vaccination history was unknown. He had been smoking tobacco for more than 20 years, with 10 cigarettes per day, and drinking alcohol for more than 20 years, with an average of 50–100 g/day.

Computerized tomography (CT) examination revealed that his 4th, 5th, and 6th left ribs and left clavicle were fractured. The planned open reduction and internal fixation of the left clavicle was scheduled on March 21, 2018. The patient’s preoperative examination suggested consciousness, with heart rate (HR) at 95 beats/min, blood pressure at 133/94 mmHg, breathing rate at 16 times/min, and body temperature at 36.4°C.

Anesthesia was induced with 4 mg of midazolam, 30 µg of fentanyl, 20 mg of etomidate, and 13 mg of cisatracurium. Sevoflurane inhalation (2–3%) and IV dexmedetomidine (1 µg/kg) were given to maintain anesthesia. During the operation, a left subclavian incision was made, and the skin and subcutaneous tissues were incised to expose the clavicle and the fracture. After subperiosteal dissection of the soft tissue, the broken end of the fracture was reduced and fixed with a screw. The clavicle was then fixed with an 8 hole steel plate and anchored with six screws. The radiograph showed good fracture reduction and good internal fixation. The operation ended at 12:05 p.m. End-tidal carbon dioxide (EtCO_2_) and temperature were monitored throughout the surgery to ensure that EtCO_2_ was between 30 and 34 mmHg and the temperature around 36.2°C.

After 25 min of the surgery, the patient was awake and transferred to the resuscitation room for continued observation. He felt chest tightness and poor breathing 65 min after surgery, and his oxygen saturation (SPO_2_) rose from 92 to 98% after inhaling oxygen. The patient was reintubated for ventilator-assisted ventilation 115 min postoperatively when he became dyspneic and his oxygen saturation dropped again. Since pulmonary embolism was a common serious complication during the surgery, C-arm and CT examinations were performed immediately. Chest CT revealed varying degrees of compressive atelectasis in both lungs and poor air content in some lung tissues. The possibility of coinfection or pulmonary contusion could not be excluded. Chest CTA showed normal, and pulmonary embolism was ruled out. Then the patient was transferred to the intensive care unit for further treatment. Endotracheal intubation and continuous invasive ventilation were applied again. The blood pressure dropped to 69/48 mmHg, and elevated pulse (150 beats/min) appeared at 5:10 p.m. Disseminated intravascular coagulation (DIC) was revealed by the D-dimer test (prothrombin time: 15.3 s, plasma prothrombin time: 14.4 s, D-dimer concentration: 11.65 μg/mL, fibrin (protofibrils) degradation product concentration: 41.47 μg/mL). Therefore, pulmonary infection, atelectasis, and DIC were considered and treated accordingly. The body temperature increased to 38.4°C for the first time at 10:00 a.m. the next day and continued to rise to 39.2°C at 6:00 p.m.

The patient was considered to develop a septic shock and was treated with anti-infective imipenem and anti-inflammatory hydrocortisone, but no significant improvement was observed. The patient was in a critical condition, blood test suggested coagulation dysfunction, and the diagnosis of DIC was considered. Therefore, the patient was given a cryoprecipitate to supplement fibrinogen, coagulation factor, and fresh frozen plasma to improve the coagulation function. The patient still suffered from circulatory instability and poor oxygenation and continued to receive norepinephrine and epinephrine to maintain blood pressure. He was also supported by extracorporeal membrane oxygenation treatment to correct internal environmental disorders (acidosis and severe hypokalemia), and continuous renal replacement therapy. The patient’s body temperature was still as high as 40°C, with intermittent chills. Auxiliary examination showed a white blood cell count at 17 × 109/L, a plasma procalcitonin concentration at 100 ng/mL, and increased inflammatory indicators. Tienam and vancomycin were applied for anti-infective treatment, but the therapeutic effect was still poor. In the end, the patient developed ventricular fibrillation and died after an unsuccessful rescue on March 24, 2018.

As the patient had developed unexplainable refractory shock, dyspnea, high fever, and hypoxemia after anesthesia, he had a very poor prognosis and anti-infection treatment was ineffective. In reviewing the patient’s disease progression, the earliest signs were not raised EtCO_2_, elevated body temperature or muscle rigidity, but rather dyspnea and low oxygen saturation. Continuous invasive mechanical ventilation could mask elevated EtCO_2_, whereas sufentanil, propofol, midazolam analgesic sedation, and vincristine muscle relaxation might cover the patient’s muscle rigidity. When the patient experienced dyspnea 115 min after surgery, myoglobin concentration increased from 198 to 605 μg/L, phosphocreatine kinase isoenzyme concentration increased from 30 to 35 U/L, and the levels of lactate dehydrogenase, hydroxybutyrate dehydrogenase, phosphocreatine kinase, adenosine deaminase, and troponin were elevated, while brain natriuretic peptide was normal (61.1 pg/mL). These changes might not have been caused by infection and heart failure but by rhabdomyolysis. On postoperative day 2, the patient was found to have elevated lactate (Lac: 6 mmol/L), increased HR (150 beats/min), decreased renal function (creatinine: 208.8 μmol/L), reduced serum potassium (K^+^ concentration: 2.9 mmol/L), and persistent hyperthermia. In conjunction with elevated myoglobin, the possibility of MH was considered.

After obtaining the consent of the patient’s family, whole-exome analysis using next-generation sequencing was performed. In the analysis, 4,813 exon regions (approximately 30 Mb) from genes known to be associated with clinical phenotypes were captured and sequenced, and the reads were successfully mapped to the human reference genome (UCSC hg19). After filtering based on sequence quality and plot score, the percentages of bases with a Qphred score of >20 or >30 were 97.15 and 93.23%, respectively. A total of 18,575 single nucleotide polymorphisms (SNPs) were identified, including 4,047 synonymous mutations, 12,374 missense mutations, 1,012 insertions, and 1,142 deletions, which included 37 nonsense mutations, 36 transcoding mutations, and 51 splice-site mutations. SNPs and indels were detected within the patient’s genome with a Het/Hom rate of 1.5.

**Figure 1:**
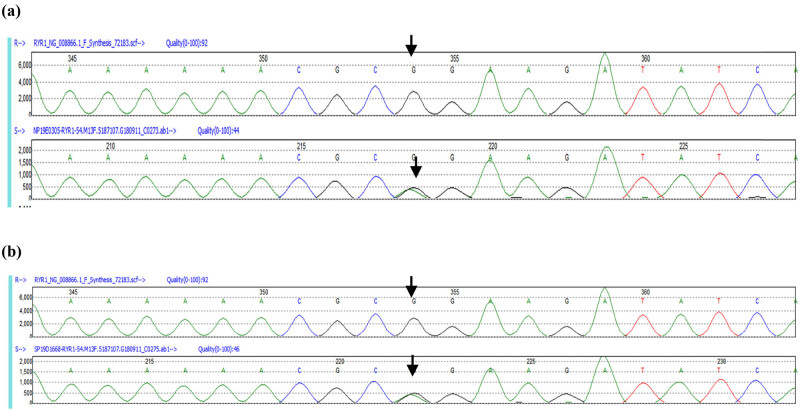
(a) The mutation in the RYR1 gene was confirmed by sequencing (c.8519G>A, p.arg2840gln). The reference sequence is at the top, and the patient’s sequence is at the bottom. (b) The mutation in the RYR1 gene of the patient’s daughter was confirmed by sequencing (c.8519G>A, p.arg2840gln). The reference sequence is at the top, and the sequence of the patient’s daughter is at the bottom.

**Figure 2:**
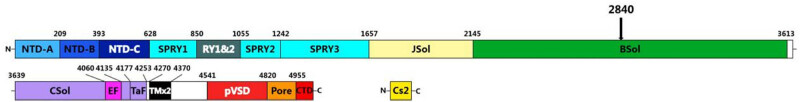
The planar structure of the RyR1 protein. Although the structural regions contained within NTD, Nsol, Bsol, and Csol are far apart in sequence, they are close in the 3D space in the context of the tetramer, allowing long-range cooperativity. Amino acid 2840 is located in the BSol domain.

**Figure 3:**
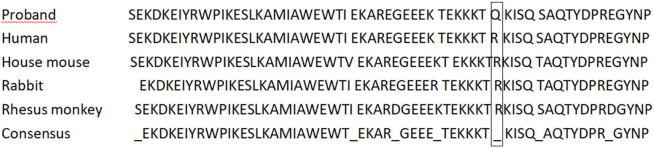
Alignment of RyR1 protein sequences. The alignment shows the comparison of the RyR1 protein sequence of the proband with that of other animals.

As shown in Figure 1a, targeted analysis of the genes known to be involved in MH identified 14 SNPs in RYR1, including six synonymous variants, seven missense mutations (two in exon regions and five in intron regions), and one missense mutation (codon 2840, c.8519G>A, p.arg2840gln, REVEL 0.47, SIFT 0) in the heterozygous form. The 1,000-genome (1000g2015aug_ALL), ExAC, and gnomAD datasets representing the normal population did not include this mutation. Importantly, the mutation in codon 2,840 was classified as a variant of uncertain significance, which is located in the Bsol structural region of RYRI (Figure 2). Bioinformatic analysis predicted that the mutation was located in the conserved region of the RYR1 gene. It is highly similar in mammals (Figure 3), including house mouse (NP_033135.2), rabbit (NP_001095188.1), and rhesus monkey (XP_028695846.1). The patient daughter’s genome was also sequenced and the same genetic mutation was identified (Figure 1b).

## 3 Discussion

In this case report, the patient was in good health before surgery and had no relevant family history, but after anesthesia, he had a sustained high fever, hypoxemia, and ventricular fibrillation that could not be relieved after potent anti-infection treatment. The condition progressed fast, and the prognosis was very poor. The earliest signs of the patient included dyspnea, decreased oxygen saturation, and low blood pressure. As the disease progressed, he developed symptom of hyperthermia, and had elevated levels of myoglobin, phosphocreatine kinase, and phosphocreatine kinase isoenzymes. As the patient received sevoflurane anesthesia, we considered the possibility of MH, and genetic testing was conducted.

Genetic testing showed a mutation in the RYR1 gene, the most common mutated gene in patients with MH (around 50%), and his daughter also carried the same mutation, which made this case highly suspicious of MH. In addition, the mutation p.arg2840trp, which affected the same amino acid residue as in the case reported here, has been previously detected in MH patients [10].

There are two widely used diagnostic approaches for MH susceptibility, including the caffeine halothane contracture test [11] recommended by the North American Malignant Hyperthermia Group, and the *in vitro* contracture test (IVCT) according to the European Malignant Hyperthermia Group [12]. However, IVCT is expensive and only available in a few hospitals in China. In the meantime, the incidence of MH is low, so the clinical promotion of IVCT is limited. The clinical grading scale (CGS) is still widely used in MH diagnosis, and a CGS score ≥50 almost confirms the diagnosis of MH [13]. An assigned CGS rank may underestimate the diagnosis of mild MH and undermonitored MH [4,14]. MH is essentially a severe reaction to certain anesthetic drugs. It is also an autosomal dominant disorder, which is associated with mutations in RYR1 and CACNA1S genes [2]. Genetic testing offers an alternative to IVCT. It has been reported that 50–70% of MH susceptibility is linked to RYR1, and over 400 MH-related variants of this gene have been identified through genetic testing [12].

The RYR1 gene encodes the skeletal muscle ryanodine receptor, which serves as a calcium release channel of sarcoplasmic reticulum as well as a bridging structure connecting the sarcoplasmic reticulum and transverse tubule [15]. The protein RyR1 is a four-fold symmetric tetramer, just like a mushroom. It contains the N-terminal domain (NTD, residues 1–392), N-terminal solenoid (Nsol, residues 393–627), bridging solenoid (Bsol, residues 2,145–3,613), and core solenoid (Csol, residues 3,667–4,174) regions [16]. When RYR1 binds with Ca^2+^, ATP, and caffeine, it results in four distinct conformations: two open states and two closed states [17]. The conformational change between open and closed states involves concerted movement of the channel core and the cytosolic shell, where the rigid unit mainly comprises shell residues 1,657–3,613 (JSol and BSol-RY3&4). In addition, variants collected in public databases are enriched in the Bsol region, which is important for maintaining effective inter-subunit interactions and channel gating. Moreover, variants in the same domain are associated with a clinically severe phenotype [18]. The variant identified in the present case was located in the Bsol region.

The patient underwent whole-exon sequencing to identify the mutations in the RYR1 gene. Mutations in the RYR1 gene can result in the King-Denborough syndrome, central hole disease, rhabdomyolysis, tachycardia, atypical periodic paralysis, and MH [19,20]. More than 150 point mutations were found in the RYR1 gene of this patient, including three “hot spots” at the n-terminal (exon 1–17), center (exon 39–46) [10], and c-terminal (exon 90–104) regions. To date, 42 missense mutations in the cDNA coding region of the RYR1 gene have been shown to be related to the occurrence of MH [21]. In this case report, a heterozygous missense mutation (codon 2,840, c.8519G>A, p.arg2840gln, REVEL 0.47, SIFT 0) was identified, which was not found in the normal population database including the 1000g2015aug_ALL, ExAC, and gnom AD datasets. If a patient carries a mutated allele of the RYR1 gene, the anesthetist should consider using other anesthetics instead of sevoflurane. Moreover, as genetic alternations are inheritable, it is recommended that his/her family members undergo screening for causative mutations. MH is an autosomal dominant disorder, and the offspring of MH patients have a 50% risk of developing this disease. Therefore, after fully informing that the test results may affect access to health insurance, employment opportunities, and even marriage [22], the patient’s daughter underwent genetic testing and the result confirmed that she had the same mutation.

Dantrolene is the only drug known to specifically treat MH [2,23]. Unfortunately, dantrolene sodium was not approved by CFDA until July 2020. Furthermore, the drug is expensive and difficult to preserve, and only a few hospitals have its stocks in China. Therefore, in most cases, when MH progresses, patients cannot receive dantrolene treatment in time. This reminds us that it is necessary to popularize and strengthen the knowledge of MH. Patients with rapidly rising or abnormally high postoperative body temperature should be alerted to the possibility of MH, and timely differential diagnosis and early treatment should be performed.

In conclusion, during orthopedic surgery, patients receiving inhaled anesthetics can exhibit MH. The patient reported here had a mutation in the RYR1 gene at position c.8519G>A (p.arrg2840gln), which might be responsible for the development of MH. His daughter also carried this mutated locus, and attention must be paid to MH prevention in her future treatment. The findings of this case report highlight that unexplained hypercarbia in arterial blood gas analysis is not easy to cover up, and the possibility of MH needs to be investigated if encountered. All anesthesiology departments should have manual procedures for handling MH reactions and anesthesia management for high-risk groups and maintain vigilance when MH triggering drugs are administered.
